# Social Disparities in Children’s Respiratory Health in El Paso, Texas

**DOI:** 10.3390/ijerph110302941

**Published:** 2014-03-11

**Authors:** Sara E. Grineski, Timothy W. Collins, Paola Chavez-Payan, Anthony M. Jimenez, Stephanie Clark-Reyna, Marie Gaines, Young-an Kim

**Affiliations:** 1 Department of Sociology and Anthropology, University of Texas of El Paso, 500 West University Ave, El Paso TX 79968, USA; E-Mails: twcollins@utep.edu (T.W.C.); paolachavez95@gmail.com (P.C.-P.); seclarkreyna@miners.utep.edu (S.C.-R.); 2 Department of Sociology, University of Minnesota (Twin Cities), Social Science Building, 267 19th Ave. S., Minneapolis, MN 55455, USA; E-Mail: jimen033@umn.edu; 3 Department of Social Work, Fayetteville State University, 1200 Murchison Rd, Fayetteville, NC 28301, USA; E-Mail: marie.gaines30@yahoo.com; 4 Department of Criminology, Law and Society, University of California Irvine, 2340 Social Ecology II Irvine, CA 92697, USA; E-Mail: youngank@uci.edu

**Keywords:** asthma, children, social disparities, respiratory health, El Paso, Texas

## Abstract

The objectives of this study were to assess prevalence of children’s respiratory health conditions and to measure and describe social disparities in children’s respiratory problems and access to health resources for asthma/wheezing management. Data were collected through a cross-sectional, observational mail survey of all primary caretakers of 4th and 5th grade children in El Paso Independent School District (El Paso, TX, USA). 6295 primary caretakers received surveys at their home address and 1904 surveys were completed and returned for a 30% response rate. El Paso children have high rates of asthma (17%) and allergies (51%). In terms of social disparities, children that are male, not poor, obese, Hispanic, born in El Paso, have a US-born caretaker, and have a caretaker who has lower levels Spanish proficiency have increased odds of respiratory problems. Among children with asthma and wheezing, disparities exist in access to care; those that are poor, with a Spanish-speaking caretaker, or with a foreign-born caretaker had increased odds of seeking care in urgent care center, emergency rooms and hospitals. Results have scholarly and practical implications for broader trends in terms of increasing prevalence of respiratory health problems across multiple scales (from El Paso to the US context to worldwide) and health disparities experienced within the rapidly growing US Hispanic population.

## 1. Introduction

Over the past decade, recognition of the importance of addressing social determinants of ill health has spread worldwide. This growing awareness was reflected in the 2005 establishment of the Commission of Social Determinants of Health by the World Health Organization [1] and has been mirrored in the Region of the Americans, where there is a strong regional movement to address social disparities in health. Related to this, we catalog rates of respiratory health problems among children living in El Paso (Texas), a US-Mexico border metropolis, assess social disparities in those problems, and then turn our focus to examining disparities in access to health care for children with respiratory health problems. These data come from a comprehensive assessment of children’s respiratory health in El Paso conducted by the Hispanic Health Disparities Research Center in 2012 through its Environment Core. The Hispanic Health Disparities Research Center, a P20 Center of Excellence, was funded by the National Institutes of Health (NIH) to address the critical health problems faced by the US-Mexico border population, which demand a systematic approach for examining health disparities.

More generally, the US-Mexico border region provides an ideal laboratory for examining social disparities in health in predominately Hispanic majority communities. This is important given the rapid growth occurring in the US Hispanic population. The border region (defined as stretching 100 km on either side of the international boundary) is disproportionately inhabited by people of Hispanic ethnicity (on the US-side, 52% are Hispanic as compared to 15% in the rest of the US). Border residents have lower levels of educational attainment, lower incomes, higher rates of unemployment, and higher poverty levels than the US population as a whole [2]. These socio-economic conditions are linked with higher rates of diabetes, obesity, tuberculosis, respiratory disorders, hepatitis B and C, and HIV/AIDS, as well as lower rates of health insurance coverage and less access to health care facilities [2]. Specifically related to children’s respiratory health, the focus of this paper, rates of hospitalizations for asthma among Texas children were 36% higher for those living in border counties as compared to children living off the border. The disparities were even greater for Hispanic children, who faced a 64% increase in odds of hospitalization for asthma if they lived in the border region, as opposed to off the border [3].

These high rates of asthma hospitalization for Hispanic children in the border region [3] are somewhat surprising, given the presence of a Hispanic Health Paradox in the United States [4]. The paradox refers the fact that in the US, the health of Hispanics, especially those of Mexican-origin, is favorable relative to other minority groups [5,6,7]. However, acculturation to US culture tends to erode the initial health advantage that immigrants have. Factors explaining the relative health advantages of Hispanic immigrants may include cultural practices, family support systems, selective migration, diet, and genetic heritage [4], but a definitive explanation has yet to be determined and recent research has begun to question whether all Hispanic subgroups enjoy the same health advantages [7,8].

### Research Questions

The paper addresses four research questions using data from El Paso children (previously introduced):

1. What percentages of children have experienced respiratory health problems (including diagnosed asthma, wheezing, bronchitis, pneumonia and allergies)?
2. Are there social disparities in these respiratory health problems? Which social groups face higher risks?
3. For those with asthma and/or wheezing, what percentages have access to different types of health care?
4. Are there social disparities for children with asthma and/or wheezing in terms accessing different types of health care?

## 2. Data and Methods

### 2.1. Data Collection

Data were collected through a cross-sectional, observational mail survey that was approved by our university’s Institutional Review Board. The closed-ended questionnaire was sent to all primary caretakers (parents and guardians) of 4th and 5th graders attending school in the El Paso Independent School District (EPISD). Surveys were conducted using the tailored design method (TDM) to obtain the highest achievable response rates by personalizing communication, following-up with non-respondents, and offering incentives [9]. All survey materials were provided to households in English and Spanish. Mailings were sent in three waves during May of 2012. Ultimately, 6295 primary caretakers received surveys at their home address and 1904 surveys were returned for a 30% response rate. Research indicates that similar and substantially lower survey response rates can yield representative samples [10,11,12,13]. The first mailing consisted of the survey packet, which included a consent letter and the survey (in both English and Spanish), a $2 incentive and a postage-paid return envelope. A week later, we mailed a bilingual reminder postcard. One week after that, we re-sent the survey packet to all non-respondents (again with $2 and a postage-paid return envelope).

### 2.2. Study Population

All respondents live in El Paso County, Texas, which had an estimated population of 827,398 in 2012. El Paso is comprised of a highly heterogeneous Hispanic population. According to the US Bureau of the Census, in 2011, 81% of its residents were Hispanic (compared with 17% for the US and 38% for TX), while smaller percentages were non-Hispanic white (14%) and non-Hispanic black (4%). El Paso County had a lower median household income (2011 US $36,333) than the State of Texas (2011 US $49,391) and the US (2011 US $50,502) with a poverty rate of 24 percent, which is higher than the national rate (16%). In 2011, just 26% of El Paso County residents spoke only English, while 72% spoke Spanish. Furthermore, 27% of the county’s Spanish speaking households did not speak English very well, 26% of county residents were foreign-born, and 15% were not US citizens.

Each survey respondent was the primary caretaker of a 4th and 5th grader enrolled in an EPISD elementary school. The EPISD is the largest school district in Region 19 (El Paso and Hudspeth Counties) of the Texas Education Agency's Educational Service Center. With more than 64,000 students across 94 campuses, the EPISD also is the 10th largest district in Texas and the 61st largest district in the United States. Within the EPISD, 83% of students are of Hispanic origin and 37% are enrolled in 58 elementary schools [14]. Children in the 4th and 5th grade from all 58 schools are represented in the dataset.

Respondents were primarily mothers (82%), with the next largest shares being fathers (10%) and grandparents (4%). More detailed descriptive statistics are presented in the Data section of this paper, but it is notable that the mean income of the surveyed households was $20,000–$29,000 in 2012. The vast majority of children (90%) were born in the United States, and 16% were not continuously covered by health insurance over the past 12 months. This approximates the high rate of uninsurance for children in Texas, which leads the nation at 19% (the US average is 9%) [2]. Ages of children in the sample ranged from 9 to 13 years with approximately 90% of them being between 9 and 11 years old.

Descriptive statistics indicate that our sample of fourth and fifth graders is generally representative of the EPISD student population across all grades, kindergarten through twelve. The percent male and percent Hispanic are nearly identical between the sample and the EPISD (49.9% *vs.* 51.4% and 82.2% *vs.* 82.6% respectively); the sample has a lower percentage of economically disadvantaged children than the EPISD as a whole (60.4% *vs.* 71.1%) [14].

### 2.3. Respiratory Health Outcomes

We define respiratory health problems for this paper to include more than just asthma, which has received sole focus in many studies. Our definition includes current wheeze, diagnosed asthma, possible undiagnosed asthma, allergies/hay fever, bronchitis, and pneumonia, which were measured based on survey items from the Phase 2 International Study of Asthma and Allergies in Childhood (ISAAC) questionnaire, the National Asthma Survey [15,16], and a previous study conducted in the EPISD [17,18]. These variables are used to address research questions 1, 2 and 4, and information about them is presented in Table 1.

**Table 1:** Respiratory health problems: survey questions, justification and coding.

Variable	Survey Question	Justification	Coding
Current Wheeze	Has your child had wheezing or whistling in the chest in the last 12 months?	Used as an asthma symptom prevalence indicator [19]	1 = yes and 0 = no

Diagnosed Asthma	Has the child ever been told by a doctor or other health professional that he or she had asthma?	Most common chronic diseases of childhood [20]	1 = yes and 0 = no

Possible Undiagnosed Asthma	(1) Has your child had wheezing or whistling in the chest in the last 12 months?	Limited access to care can lead to under diagnosis of asthma in symptomatic children [20]	1 = positive response for Current Wheeze and a negative response for Diagnosed Asthma and 0 if not.
	(2) Has the child ever been told by a doctor or other health professional that he or she had asthma?		
Allergies/Hay fever	“Has your child ever had allergies or hay fever?”	Growing rates worldwide with high rates in the study community [18]	1 = yes and 0 = no
Current Bronchitis	During the past 12 months, was the child diagnosed with bronchitis by a health care provider?	Common childhood occurrence that receives less attention than asthma; frequent infections associated with poverty [21]	1 = yes and 0 = no
Current Pneumonia	During the past 12 months, was the child diagnosed with pneumonia by a health care provider?	See Current Bronchitis justification	1 = yes and 0 = no

### 2.4. Social Disparity Indicators

We selected seven social disparity indicators, based on a review of the literature, which are used to address Research Questions 2 and 4. Information about them is presented in Table 2. The demographic questions were taken from the American Community Survey and US Census and the question about El Paso residence came from a previous EPISD study [17].

**Table 2:** Social disparity indicators: survey questions, justification, coding and descriptive statistics.

Variable	Survey Question	Justification	Coding	%
Child is Male	What is the sex of the child?	Boys were more like to be diagnosed with asthma and to wheeze than girls [22]	1 = male and 0 = female	50% male
Household is in Poverty	(1) What is your yearly total household income for 2011 before taxes?	Poor children had higher asthma prevalence [23]	Re-coded based on US poverty guidelines for income and household size;	38% in poverty
	(2) How many people live at this address?		1 = poor and 0 = not poor	
Child is Obese	(1) How tall is the child as of now?	Associated with asthma [24] and lower airway obstruction [25]	Calculated body mass index and recoded based on age/sex specific classifications [26]; 1 = obese and 0 = not obese	21% obese
	(2) How much does the child weigh as of now?			
Child is Hispanic	Is this child of Hispanic, Latino or Spanish origin?	Hispanic ethnicity, especially Mexican-origin, was a protective factor for asthma and respiratory conditions [27]	1 = Hispanic and 0 = not Hispanic	82% Hispanic
Caretaker is US-Born	Where were you born, in the United States or outside of the United States?	Children of US-born mothers had higher rates of asthma than children of foreign-born mothers [28]	1 = US born and 0 = foreign-born	50% born in the US
Caretaker Speaks Spanish	How well do you speak Spanish, very well, well, not well, or not at all?	Mother’s preference for speaking Spanish was associated children’s with lower rates of diagnosed asthma and higher rates of undiagnosed asthma [29]	1 = speak Spanish very well and 0 = less than very well	55% speak Spanish very well
Child was Born in El Paso	How long as this child lived in El Paso County, from “since birth” up to “for less than 12 months?	Rates of allergies and asthma in El Paso children in 1999 were significantly higher for those born there [18]	1 = born in El Paso and 0 = born elsewhere	54% born in El Paso

### 2.5. Access to Care Variables

These variables are used to address Research Questions 3 and 4 and were asked only of caretakers with children who have ever wheezed or been diagnosed with asthma. Information about them is presented in Table 3. These survey questions were taken from Phase II of the ISAAC and a previous EPISD study [17].

**Table 3:** Access to care variables: survey questions, justification and coding.

Variable	Survey Question	Justification	Coding
Medicated	In the past 12 months, has your child used any medicines, pills, inhalers/puffers or other medications for wheezing or asthma?	Children with asthma or wheezing may take medications to better control their symptoms [30]	1 = yes and 0 = no
Hospitalized	In the past 12 months, how many times has your child been admitted to the hospital because of wheezing or asthma, none, one time, two times or more than two times?	Hospitalizations can be proxies for uncontrolled asthma, since the condition can usually be managed without hospitalization [30]; they also represent serious cases of asthma or wheezing	Recoded as 1 = yes (1 or more times) and 0 = no (none)
Insured	Was the child continuously covered by medical insurance over the last 12 months?	Lacking health insurance has been associated with discontinuity of care, inadequate access to needed medications, and delayed treatment for asthma [31]	1 = yes and 0 = no
Source of Care:	In the past 12 months, did the child visit any of the following health professionals for a wheezy episode: school nurse; doctor in a clinic; respiratory specialist; doctor at an urgent care center; hospital emergency department?	Children seek care for wheezing/asthma attacks in a variety of places [32,33]	1 = yes and 0 = no for each source of care
—School Nurse			
—Clinic			
—Specialist			
—Urgent Care			
—Emergency Room			

### 2.6. Multiple Imputation of Missing Values

To address nonresponse bias, the missing values of all analysis variables were multiply imputed. Multiple imputation (MI) is currently a best practice for addressing missing data in statistical analysis. MI involves creating multiple sets of values for missing observations using a regression-based approach. It is used to avoid the bias that can occur when missing values are not missing completely at random (MCAR) [34] and is appropriate for self-reported survey data [35]. In SPSS, 20 imputed datasets were specified to increase power and 200 between-imputation iterations were used to ensure that the resulting imputations were independent of each other [35]. Using 20 datasets is the current “rule of thumb” in multiple imputation as it maximizes power (as opposed to using 3–5 datasets, which used to be the convention) and improves the validity of multi-parameter significance tests [35]. Analyzing a single imputed dataset would effectively treat the filled-in values as real data, so even the best imputation technique, when used with just one imputed dataset may underestimate sampling error. Multiple imputation techniques appropriately adjust the standard errors for missing data [35]. We ran multiple imputation twice, once with all respondents for research questions 1 and 2, and then again with only the children with reported wheezing and/or asthma for research questions 3 and 4. The percent missing for the variables ranged from a low of 2.7% (Current Wheeze) to a high of 26.4% (Obesity).

### 2.7. Analysis Methods

To answer research questions 1 and 3, we reported pooled mean values for each variable. Mean values are the only uni-variate statistic reported by SPSS when analyzing multiply imputed data (e.g., the standard deviation is not provided). To answer research questions 2 and 4, we used unadjusted odds ratios to compare social groups. Using SPSS, we ran cross-tabs and used the pooled results to calculate the odds ratio statistics. Results of the bi-variate analyses allow us to generally characterize basic social disparities in respiratory health problems and access to health care among El Paso children; the purpose is not to evaluate the relative importance of multiple determinants of respiratory health problems or access to care by testing them in multivariate models.

## 3. Results

### 3.1. Research Question 1

We found that 15% of children had current wheeze and 17% had been diagnosed with asthma at some point during their lifetime; 7% had possible undiagnosed asthma. Over half (51%) had hay fever/allergies. In terms of respiratory infections, 10% had bronchitis in the past year and only 1% had pneumonia. Because so few children had pneumonia, it was excluded from further analysis.

### 3.2. Research Question 2

Analyses revealed many social disparities in respiratory health problems among El Paso youth (see Table 4).

**Table 4:** Social disparities in respiratory health outcomes for all children: odds ratios and statistical significance.

Respiratory Outcome	Male ^a^	Poor ^b^	Obese ^c^	Hispanic ^d^	US-Born Caretaker ^e^	Caretaker speaks Spanish “very well” ^f^	Birth in El Paso ^g^
Current Wheeze	1.52 **	0.70 **	1.30 **	1.10	1.76 **	0.74 **	1.53 **
Diagnosed Asthma	1.62 **	0.82 *	1.36 **	1.51 **	1.40 **	0.66 **	1.21 *
Possible Undiagnosed Asthma	1.47 **	0.86	1.25	0.87	1.43 **	0.97	1.28
Allergies/Hay Fever	1.15 *	0.57 **	0.91	1.47 **	2.10 **	0.60 **	1.36 **
Bronchitis	1.15	1.04	0.89	1.44 **	1.63 **	0.70 **	1.18

** *p* < 0.05; * *p* < 0.10; Reference Category: ^a^ Female; ^b^ Not Poor; ^c^ Not Obese; ^d^ Not Hispanic; ^e^ Foreign Born caretaker; ^f^ Caretaker speaks Spanish less than very well; ^g^ Not born in El Paso.

Boys had significantly higher odds of suffering from current wheeze, asthma, possible undiagnosed asthma and allergies/hay fever than did girls. Poverty was significantly associated with lower odds of current wheeze and allergies/hay fever. Obese children had higher odds of current wheeze and diagnosed asthma than did non-obese children. Hispanic children had higher odds of diagnosed asthma, allergies, and bronchitis than did non-Hispanic children. Children with US-born primary caretakers had significantly higher odds of all the health conditions than did those with foreign-born caretakers. Children whose caretakers spoke Spanish very well had significantly lower odds of current wheeze, diagnosed asthma, allergies, and bronchitis than those with primary caretakers did not speak Spanish very well. Lastly, children born in El Paso had significantly higher odds of current wheeze and allergies than those not born in El Paso.

### 3.3. Research Question 3

Among the 593 children who have ever wheezed or been diagnosed with asthma, 53% took prescription medications in the last year. In terms of the specific medications the children were taking (not introduced in Table 3), 29% were prescribed rescue inhalers (e.g., albuterol or Xopenex^®^ (levalbuterol)), 17% took Singulair^®^ or another montelukast, 15% were on an inhaled corticosteroid like Flovent^®^ (fluticasone) or Pulmicort^®^ (budenoside), 8% took long-acting bronchodilators like Serevent^®^ (salmeterol), 12% took long-acting bronchodilators in combination with a corticosteroid like Advair^®^ (fluticasone/salmeterol), and11% had been prescribed corticosteroid tablets or syrups like prednisone. In the last year, 6% were hospitalized for asthma/wheezing. In terms of where they access care for wheezing, 33% went to the school nurse, 43% went to a clinic, 19% saw a respiratory specialist, 19% went to an urgent care center, and 15% went to the emergency room.

### 3.4. Research Question 4

Analyses of access to care disparities revealed reverse trends when compared to the disparities found for the respiratory health conditions (see Table 5). Poor children were more likely to have been hospitalized, to use urgent care and emergency care and to be uninsured than non-poor children. They were also 25% less likely to have taken medications for asthma (*p* < 0.10). Children with a US-born caretaker were less likely to be hospitalized and less likely to have been to the emergency room than children with a foreign-born caretaker. Children with Spanish-speaking caretakers were significantly more likely to have been hospitalized for asthma; they were less likely to be insured (*p* < 0.10). Hispanic children were less likely to be hospitalized for asthma then non-Hispanic children. There were no significant access to care disparities for sex, obesity, or birth in El Paso.

**Table 5:** Social disparities for access to care for children with asthma and/or wheezing: odds ratios and statistical significance.

	Male ^a^	Poor ^b^	Obese ^c^	Hispanic ^d^	US-Born Caretaker ^e^	Caretaker speaks Spanish “very well” ^f^	Birth in El Paso ^g^
Medicated	0.95	0.75 *	0.85	1.04	1.08	0.93	1.14
Hospitalized	1.21	2.28 **	1.09	0.34 **	0.54 **	2.47 **	0.85
School Nurse	0.76 *	0.95	0.94	1.26	1.24	0.90	1.27
Clinic Visit	1.01	1.07	1.22	0.84	1.01	1.01	0.89
Respiratory Specialist	1.16	1.27	1.22	0.90	0.94	0.95	1.17
Urgent Care Center	0.99	1.90 **	0.68	0.91	0.81	0.91	0.94
Emergency Room	1.00	1.59 **	1.99	0.58 *	0.67 *	1.33	1.16
Insured	1.18	0.50 **	1.12	2.42 **	1.38	0.62 *	0.99

** *p* < 0.05; * *p* < 0.10; Reference Category: ^a^ Female; ^b^ Not Poor; ^c^ Not Obese; ^d^ Not Hispanic; ^e^ Foreign Born caretaker; ^f^ Caretaker speaks Spanish less than very well; ^g^ Not born in El Paso.

## 4. Discussion

Perhaps the most important findings to come from this study are those documenting that current respiratory illness rates among El Paso children are high in relation to rates documented through prior research in the study area and in comparison to documented US rates. In a study of 4th and 5th graders in EPISD schools conducted in 1999 [17], children had lower rates of asthma (11%), allergies (31%) and bronchitis (7%) than they did in 2012. Compared to children in the US, El Paso’s children seem to suffer disproportionately from respiratory illnesses. Lifetime asthma rates for El Paso children are 1.3 times the national average for children (13%) [36], which is surprising given that over 80% of surveyed children were Hispanic with 69% being of Mexican origin; at a national level, Hispanic and especially Mexican-origin populations have lower rates of asthma than non-Hispanics. Rates of allergies/hay fever were also elevated. El Paso children had rates of allergies/hay fever that vastly exceeded the US average for hay fever for children under 17 (10%) [37]. These high rates may relate to the urbanized, windswept desert environment of El Paso. Previous studies in the area have demonstrated respiratory health effects in children from traffic and non-traffic related pollution [38] and dust storms [39].

Two anomalous findings from our analyses include the increased risk of respiratory health problems for Hispanic children and for children not in poverty (Table 4). First, the increased risk for Hispanic children (*i.e.*, they were 51% more likely to have diagnosed asthma, 47% more likely to have allergies, and 44% more likely to have had bronchitis) is surprising as Mexican-origin children (the majority of the children in this study) tend to have lower rates of asthma than non-Hispanic whites and blacks [36] and because non-Hispanics had higher rates of asthma and allergies as compared to Hispanics in the comparable population (El Paso 4th and 5th graders) in 1999 [18]. The higher occurrence of bronchitis among Hispanic children may be related to increased drug-resistance, due to increased use of antibiotics, accessed without a prescription in Mexico [40,41]. For Hispanic children with asthma, there were few disparities in access to care when compared to non-Hispanic children, only a reduced likelihood of hospitalization.

Second, poverty was significantly associated with a 30% reduction in the odds of current wheeze and 43% reduction in the odds of allergies/hay fever, which is counterintuitive from a social disparities perspective (Table 4). While asthma is often associated with poverty [23], other studies have found no significant relationship between lower socioeconomic status and diagnosed asthma [42,43]. In El Paso, the protective effects of poverty might be linked to a Hispanic health paradox, given that foreign-born residents are highly likely to be poorer than US-born residents. This is also supported by a finding from an earlier study which showed that longer length of residence in El Paso was associated with the development of allergies in EPISD school children [18]. The association between poverty and lower odds of allergies may relate to the presence of less sterile home environments, as there is some evidence, as per the hygiene hypothesis, that exposure to microbial compounds found in dirt can be healthy for one’s immune system [44]. However, while poverty may be protective for developing respiratory problems, it is a risk factor for poor asthma/wheezing management (Table 5). Specifically, poor children were 128% more like to have been hospitalized, 90% more likely to have used urgent care, 59% more likely to have visited the emergency room, 50% less likely to be insured, and 25% less likely to have taken medications for asthma/wheezing.

The findings for the caretaker’s nativity and caretaker’s Spanish-proficiency as related to the odds of having a respiratory health problem (Table 4) also seem to reflect a Hispanic health paradox (given that 82% of the children are Hispanic) whereby lower levels of acculturation (*i.e.*, being foreign-born and speaking Spanish very well) are associated with reduced odds of illness. The protective effect of a foreign-born caretaker (also found in [28]) cannot be related *only* to a lack of access to health care (and thus reduced likelihood of an asthma diagnosis) because we find it for both diagnosed asthma and possible undiagnosed asthma. We found that when the caretaker is proficient in Spanish, the child is at lower risk of a host of respiratory health conditions; they were 26% less likely to have wheezed, 34% less likely to have been diagnosed with asthma, 40% less likely to have had allergies, and 30% less likely to have had bronchitis.

Research has demonstrated that a mother’s preference for speaking Spanish, as opposed to English, was associated with lower rates of diagnosed asthma and higher rates of asthma symptoms without a diagnosis for children [29]. In El Paso, the odds ratio for possible undiagnosed asthma variable was not significant, although the results suggests risk 3% was higher for those not speaking Spanish very well. The protective health effects of lower acculturation might be due to strong familial, social and economic ties which exist amongst immigrant groups or an avoidance of American health behavioral norms (e.g., abstinence from smoking and/or maintenance of healthier diets) [45]. It is important to note that, in contrast to many US localities, El Paso’s location along the Mexican border situates residents in a region where many health care services are offered in Spanish by Spanish-speaking professionals or by assistants and technicians serving as informal interpreters [46].

However, findings indicate that a lack of acculturation is not protective once the child has asthma/wheezing. Children with a foreign-born caretaker were more likely to be hospitalized and to have been to the emergency room for asthma/wheezing, while children with a Spanish-speaking caretaker were 147% more likely to have been hospitalized and 58% less likely to have health insurance (Table 5). These findings likely reflect poor access to asthma management resources among immigrant, Spanish-speaking families because hospitalizations are a proxy for uncontrolled asthma, since with proper health care, the condition can typically be managed so that the child is never hospitalized [30].

Like previous studies, we found that boys, obese children, and those born in El Paso had higher odds of suffering from respiratory health problems. The increased odds of asthma for El Paso’s boys was even higher than the national average (61% higher than girls *vs.* 30% higher [36]). Obese children had 30% higher odds of current wheeze and 36% higher odds of diagnosed asthma than did non-obese children (the odds were also higher for undiagnosed asthma for obese children). Obesity and its links to respiratory problems are a growing concern, especially as evidence mounts that obesity is not only a result of asthma symptoms, but also a factor that contributes to the development of asthma and persistent wheezing [47]. Like a previous study [18], we also found that being born in El Paso was a risk factor for wheezing, diagnosed asthma and allergies, with the odds being 53%, 21% and 36% higher respectively. Svendsen and colleagues [18] argued that increased risk for El Paso’s children was due either to community-wide environmental exposures or the increased health of immigrants. The second argument parallels other work in Mexican-origin populations which has found that birth in the US increases risk of developing asthma as compared to living in Mexico during the first year of life [48].

### Limitations and Future Directions

The study uses a cross-sectional approach, which limits our ability to assess causal relationships. We also relied only on parental reports of children’s respiratory health conditions; no measures of lung function were collected as part of this study. The combination of allergies and hay fever into one survey question does not allow for the analysis of these conditions separately. We chose to combine them in order to be perfectly comparable with the 1999 survey done in the same school district [17]. Future studies in this area could employ more complex multivariate statistical models to clarify the most important covariates of the respiratory conditions under focus; additionally, in-depth qualitative interviews could be conducted to better understand underlying causes of the statistical associations observed.

## 5. Conclusions

Results have scholarly and practical implications for broader trends in terms of increasing prevalence of respiratory health problems across multiple scales (from El Paso to the US context to worldwide) and health disparities experienced within the rapidly growing US Hispanic population. The high and increasing rates of respiratory health problems documented in the study population align with recent research findings [23]. Due to climate change, respiratory health problems are expected to increase in prevalence and severity in the coming decades [49]. Thus, results indicate that more resources must be directed toward examining and addressing respiratory health problems in the context of generally amplified risks, and especially among children, who are at greatest physiological risk to adverse consequences.

Considerable attention has been placed on the relatively low asthma prevalence rate among Hispanic (particularly Mexican-origin) people in the US. However, given the rapid Hispanic population growth underway, there is an urgent need to examine respiratory health problems based on a more nuanced health disparities perspective, focused not just on asthma prevalence in the Hispanic population (compared, for example, non-Hispanic whites or blacks), but on disparities in multiple respiratory health conditions as well as access to care experienced by diverse subgroups that comprise the heterogeneous and dynamic Hispanic category. Taking a more nuanced perspective here, our analyses revealed two general patterns that paint a more complex picture of health disparities, particularly in relation to Hispanic children. One, in El Paso, which has a vast Hispanic majority, we found that Hispanic children experienced higher prevalence rates than non-Hispanics, which is the reverse of what was found in a study conducted in 1999 [17] and what has been documented at the national level [36]. Two, while some results suggest that Hispanic immigrant status (which is closely connected with caretakers’ foreign-birth/Spanish proficiency and household poverty) is protective against respiratory health problems, other results indicate that Hispanic immigrant status is simultaneously a risk factor that amplifies suffering due to respiratory health problems via reduced capacity to successfully manage asthma without using emergency rooms and urgent care centers. Certainly, regular use of these sorts of care facilities is not desirable and is indicative of poor asthma management [30]. Use of urgent and emergency care reflects insufficient primary/preventative care for some children. Specifically, low income and public insurance status have been associated with urgent care and emergency room visits for children’s asthma and use of these services represents a financial burden for patients and taxpayers [32,50,51]. To avoid increasingly disproportionate respiratory health impacts in the coming decades, targeted efforts must be committed toward increasing access to quality care, especially for recent immigrants and the poor.
